# Supplementary material for: In situ strain tuning of the metal-insulator-transition of Ca2RuO4 in angle-resolved photoemission experiments
Source: Nat Commun. 2018 Oct 31;9:4535. doi: 10.1038/s41467-018-06945-0 (PMC6208396; doi:10.1038/s41467-018-06945-0)
Supplement: Supplementary file 1 — Supplementary Information [file 41467_2018_6945_MOESM1_ESM.pdf]

Supplementary Information: *In-situ* strain tuning of the metal-insulator-transition of Ca_2RuO_4 in angle-resolved photoemission experiments

S. Riccò,[1] M. Kim,[2,3] A. Tamai,[1] S. McKeown Walker,[1] F. Y. Bruno,[1] I. Cucchi,[1] E. Cappelli,[1] C. Besnard,[1] T. K. Kim,[4] P. Dudin,[4] M. Hoesch,[4,*] M. J. Gutmann,[5] A. Georges,[2,3,1,6] R. S. Perry,[7] and F. Baumberger[1,8,†]

[1]*Department of Quantum Matter Physics, University of Geneva, 24 Quai Ernest-Ansermet, 1211 Geneva 4, Switzerland*

[2]*Centre de Physique Théorique Ecole Polytechnique, CNRS, Universite Paris-Saclay, 91128 Palaiseau, France*

[3]*College de France, 11 place Marcelin Berthelot, 75005 Paris, France*

[4]*Diamond Light Source, Harwell Campus, Didcot, United Kingdom*

[5]*ISIS Neutron and Muon Source, Science and Technology Facilities Council, Rutherford Appleton Laboratory, Didcot OX11 0QX, United Kingdom*

[6]*Center for Computational Quantum Physics, Flatiron Institute, 162 5th Avenue, New York, NY 10010, USA*

[7]*London Centre for Nanotechnology and UCL Centre for Materials Discovery, University College London, London WC1E 6BT, United Kingdom*

[8]*Swiss Light Source, Paul Scherrer Institut, CH-5232 Villigen PSI, Switzerland*

Supplementary Note 1: Single crystal neutron diffraction

Supplementary Figure 1 shows the Pr-doping dependence of the lattice parameters b and c and the orthorhombicity $1 - a/b$ at 10 K and 300 K, together with data from Friedt *et al.* [1] for Ca_2RuO_4 in the *L-Pbca* phase at 400 K. The tentative phase boundary between the insulating *S-Pbca* and the metallic *L-Pbca* phase is indicated by the background color. Clearly, increasing Pr doping drives the ground state crystal structure progressively closer to the phase boundary, in line with the suppression of $T_{\rm MI}$. Within the high temperature *L-Pbca* phase, on the other hand, we observe only small changes in lattice constants between with doping.

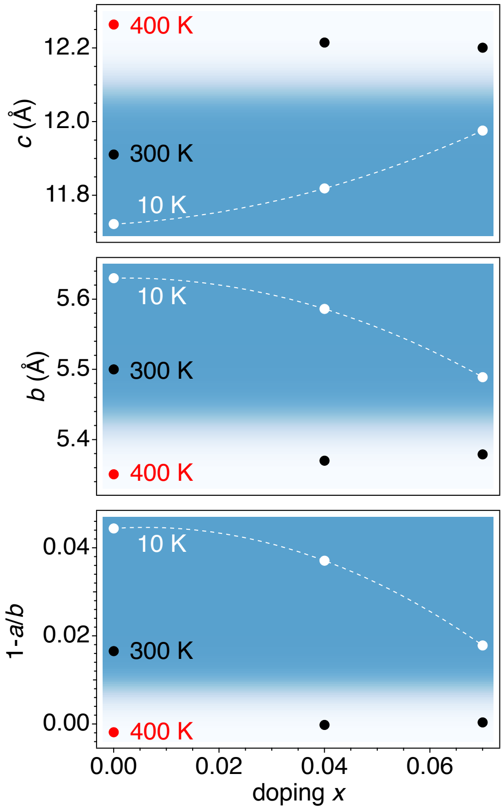

Supplementary Figure 1. Doping evolution of the crystal structure. *S-Pbca* and *L-Pbca* phases are indicated by blue and white regions. Red, black and white markers correspond to 400 K, 300 K and 10 K, respectively.

The crystal structures together with further details on data collection and refinement are reported in the CIF files available online and summarized in Tables 1-3 below.

Supplementary Table 1. Single crystal crystallographic data for $Ca_{2-x}Pr_xRuO_4$, $x = 0$, 0.04 and 0.07, at 10 K and 300 K. Lattice parameters are measured in Angstrom, while the atomic positions are given in fractional coordinates of the unit cell.

Temperature (K)		10			300		
doping x		0	0.04	0.07	0	0.04	0.07
Space group		P b c a					
a		5.379(2)	5.380(2)	5.392(3)	5.409(2)	5.3700(19)	5.377(3)
b		5.630(2)	5.5867(19)	5.492(3)	5.500(2)	5.3709(19)	5.379(3)
c		11.723(4)	11.820(4)	11.972(8)	11.910(4)	12.215(4)	12.203(8)
Volume		355.0(2)	355.3(2)	354.6(4)	354.3(2)	352.3(2)	353.0(3)
Ru	x	0.5	0.5	0.5	0.5	0.5	0.5
	y	0	0	0	0	0	0
	z	0	0	0	0	0	0
Ca (Pr)	x	0.49710	0.49490	0.49040	0.49210	0.03190	0.48970
	y	0.05870	0.05573	0.04530	0.04700	0.48800	0.03270
	z	0.35281	0.35240	0.35040	0.35160	-0.34924	0.35050
O(1)	x	0.30550	0.30470	0.30430	0.30310	0.30501	0.30580
	y	0.30030	0.30064	0.30080	0.30110	0.30470	0.30360
	z	0.02799	0.02649	0.02510	0.02386	-0.01755	0.01730
O(2)	x	0.56890	0.56630	0.55940	0.56000	-0.01155	0.54540
	y	-0.02240	-0.02131	-0.01820	-0.01830	0.54610	-0.01170
	z	0.16471	0.16492	0.16560	0.16460	-0.16563	0.16500
Ru-O(1) r_x		2.015(3)	2.0058(11)	1.983(4)	1.989(3)	1.9568(12)	1.950(3)
Ru-O(1) r_y		2.0180(17)	2.0064(12)	1.995(4)	1.991(3)	1.9546(12)	1.966(3)
Ru-O(2) r_z		1.9702(16)	1.9853(12)	2.011(4)	1.990(3)	2.0392(12)	2.029(3)
Ru-O-Ru bond (deg)		149.74(12)	150.28(10)	150.65(18)	151.37(15)	152.28(10)	152.43(15)
RuO_6 tilt (deg)		11.46(5)	10.92(6)	9.61(8)	9.83(6)	7.19(5)	7.14(7)

Supplementary Table 2. Single crystal crystallographic data for $Ca_{2-x}La_xRuO_4$, x = 0.04, 0.07 and 0.11, at 4 K, 10 K and 300 K. Lattice parameters are measured in Angstrom, while the atomic positions are given in fractional coordinates of the unit cell.

Temperature (K)		10		4	300	
doping x		0.04	0.07	0.11	0.04	0.07
Space group				P b c a		
a		5.387(2)	5.3857(19)	5.3709(19)	5.361(2)	5.378(2)
b		5.573(2)	5.512(2)	5.3808(19)	5.3716(19)	5.369(2)
c		11.833(4)	11.957(5)	12.211(4)	12.236(4)	12.239(5)
Volume		355.2(2)	354.9(2)	352.9(2)	352.4(2)	353.4(2)
Ru	x	0.5	0.5	0.5	0.5	0.5
	y	0	0	0	0	0
	z	0	0	0	0	0
Ca (La)	x	0.49454	0.49229	0.48898	0.48970	0.48980
	y	0.05422	0.04755	0.03483	0.02980	0.02980
	z	0.35197	0.35124	0.34955	0.34886	0.34994
O(1)	x	0.30500	0.30422	0.30381	0.30562	0.30440
	y	0.30074	0.30095	0.30342	0.30550	0.30310
	z	0.02614	0.02429	0.01920	0.01691	0.01682
O(2)	x	0.56535	0.56000	0.54884	0.54365	0.54320
	y	-0.02096	-0.01992	-0.01364	-0.01120	-0.01110
	z	0.16502	0.16534	0.16608	0.16558	0.16572
Ru-O(1) r_x		2.0021(9)	1.9869(11)	1.9572(8)	1.9549(12)	1.9486(18)
Ru-O(1) r_y		2.0071(8)	1.9931(10)	1.9587(8)	1.9542(11)	1.9596(18)
Ru-O(2) r_z		1.9876(9)	2.0062(11)	2.0462(9)	2.0404(10)	2.0424(18)
Ru-O-Ru bond (deg)		150.33(8)	150.99(9)	152.21(8)	152.19(10)	152.93(13)
RuO_6 tilt (deg)		10.76(4)	9.79(5)	7.65(4)	6.80(4)	6.74(5)

Supplementary Table 3. Single crystal crystallographic data for $Ca_{2-x}Nd_xRuO_4$, $x = 0.04$ at 10 K and 300 K. Lattice parameters are measured in Angstrom, while the atomic positions are given in fractional coordinates of the unit cell.

Temperature (K)		10	300
doping x		0.04	
Space group		P b c a	
a		5.372(3)	5.369(4)
b		5.585(2)	5.378(3)
c		11.821(5)	12.200(7)
Volume		354.7(3)	352.3(4)
Ru	x	0.5	0.5
	y	0	0
	z	0	0
Ca (Nd)	x	0.49430	0.49060
	y	0.05570	0.03130
	z	0.35234	0.34890
O(1)	x	0.30490	0.30510
	y	0.30000	0.30530
	z	0.02715	0.01740
O(2)	x	0.56740	0.54510
	y	-0.02160	-0.01200
	z	0.16518	0.16590
Ru-O(1) r_x		2.002(3)	1.959(3)
Ru-O(1) r_y		2.008(3)	1.956(3)
Ru-O(2) r_z		1.990(2)	2.039(3)
Ru-O-Ru bond (deg)		150.08(14)	152.20(15)
RuO_6 tilt (deg)		11.06(8)	7.06(8)

Supplementary Note 2: Localization of doped carriers in the *S-Pbca* phase

In Supplementary Figure 2 we compare the resistivity of lightly rare earth doped Ca_2RuO_4 to similar doping levels in the cuprate $La_{2-x}Sr_xCuO_4$ [2] and in the single layer iridate S_2 $_{-x}La_xIrO_4$ [3]. In the latter systems, carriers rapidly delocalize away from the stoichio-metric Mott phase, driving the system towards metallicity. In contrast, doped Ca_2RuO_4 shows a clear MIT and remains highly insulating at low temperature. As shown in Supple-mentary Figure 2, the low-temperature resistivity of $Ca_{2-x}Pr_xRuO_4$ with $x = 0.07$ is more than five orders of magnitude higher than in cuprates or iridates with comparable doping. For $x = 0.04$ the difference is even more pronounced. This provides compelling evidence for a complete localization of the extra rare earth electrons in doped Ca_2RuO_4.

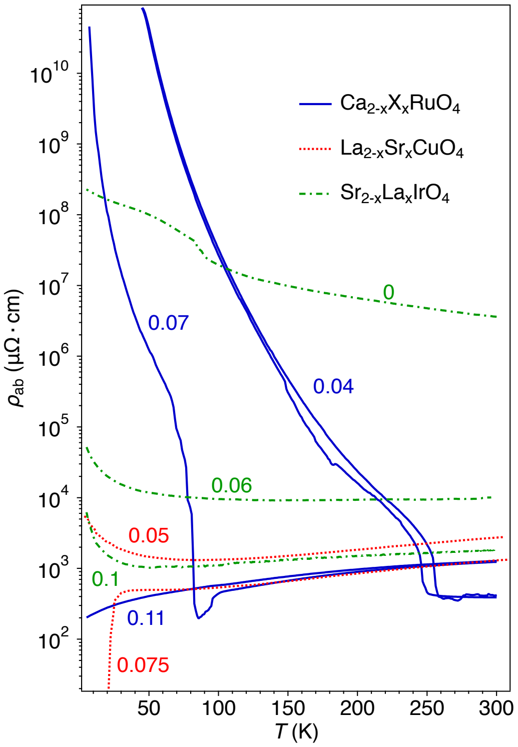

Supplementary Figure 2. Comparison of the resistivity of lightly rare earth X doped Ca_2RuO_4 (La $x = 0.11$, Pr $x = 0.04$ and 0.07) with similar doping levels in cuprates (from [2]) and iridates [3].

Supplementary Note 3: Strain evaluation

We express the nominal compressive strain on the b-axis of the sample as $\epsilon^{\mathrm{i}}_{\mathrm{xx}} = (\Delta\, L/L)_{\mathrm{CuBe}} +$ $(\Delta\, b/b)_{\mathrm{sample}}$, where $(\Delta\, L/L)_{\mathrm{CuBe}}$ is the thermal contraction of the CuBe substrate and $(\Delta\, b/$ $b)_{\mathrm{sample}} = (b^{300\,\mathrm{K}} - b^{10\,\mathrm{K}})/b^{10\,\mathrm{K}}$ is the compressive strain of $Ca_{2-x}Pr_xRuO_4$ samples

constrained to the L-$Pbca$ phase at low temperature. We use literature values to estimate $(\Delta L/L)_{\mathrm{CuBe}} \sim -0.28\%$ [4] and calculate $(\Delta b/b)_{\mathrm{sample}} \sim -3.8\%$ (-2%) for Pr doping $x = 0.04$ (0.07) from our neutron scattering data and thus find $\epsilon^{\mathrm{i}}_{\mathrm{xx}} \sim -4.1\%$ (-2.3%). Analogously, we express the compressive strain on the a-axis as $\epsilon^{\mathrm{i}}_{\mathrm{yy}} = (\Delta L/L)_{\mathrm{CuBe}} + (\Delta a/a)_{\mathrm{sample}}$, with $(\Delta a/a)_{\mathrm{sample}} = (a^{300\ \mathrm{K}} - a^{10\ \mathrm{K}})/a^{10\ \mathrm{K}}$, and calculate $(\Delta a/a)_{\mathrm{sample}} \sim -0.19\%$ (-0.28%) for Pr doping $x = 0.04$ (0.07), obtaining $\epsilon^{\mathrm{i}}_{\mathrm{yy}} \sim -0.47\%$ (-0.56%). Since the strain values along the two in-plane axes differ by nearly an order of magnitude, we treat the strain ϵ^{i} as approximately uniaxial. As shown in Fig. 1 of the main text, this strain is almost completely transmitted to the sample surface for sufficiently thin samples. In all our experiments, samples were glued to the substrates using EPO-TEK® H21D electrically conductive silver epoxy.

The initial strain is gradually released in our experiment as the $8 \times 2 \times 1$ mm^3 CuBe substrates are deformed by pressing a 1 mm diameter stainless steel ball against their lower surface. We calibrated the strain $\epsilon^{\mathrm{bend.}}$ at the surface of the substrate as a function of the vertical displacement of the ball with a finite element analysis simulation using COMSOL Multiphysics® modeling software. Supplementary Figure 3**a**,**b** show the results along the x and y directions of the substrate's reference frame for maximal vertical displacement, while Supplementary Figure 3**c** illustrates the evolution of the strain at the center of the substrate. Clearly, $\epsilon^{\mathrm{bend.}}$ is highly anisotropic, which justifies a one-dimensional treatment of the total strain $\epsilon^{\mathrm{tot}} = \epsilon^{\mathrm{i}} + \epsilon^{\mathrm{bend.}}$.

Supplementary Note 4: Phase coexistence at intermediate strain

In Supplementary Figure 4 we reproduce the angle-integrated energy-distribution curves (EDCs) for decreasing compressive strain shown in the main text. Taking the spectra at $\epsilon^{\mathrm{tot}}_{\mathrm{xx}} \sim -0.6\%$ and -1.9% as representative for the insulating S-$Pbca$ and the metallic L-$Pbca$ phase respectively, we describe the data at intermediate strain as a linear combination of these limiting cases. As shown in the figure, this approach provides a good description of the data at all strain levels, providing strong evidence for phase coexistence typical of a first order phase transition.

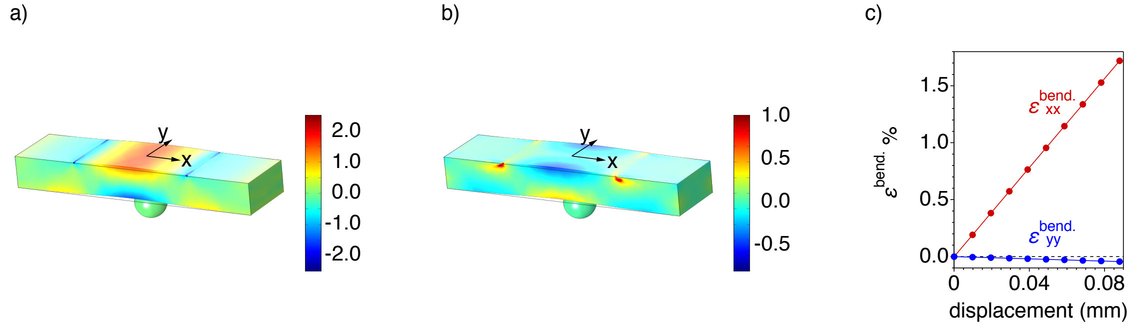

Supplementary Figure 3. **a,b** Finite element analysis simulations of the deformation of the substrates in our strain apparatus. The clamping pieces holding down the substrates have been included in the simulations but are not shown for clarity. The colors encode strain along (**a**) and perpendicular (**b**) to the bending direction. **c** Calibration of the strain ϵ_{xx} and ϵ_{yy} as a function of displacement of the stainless steel ball pressing on the CuBe substrate.

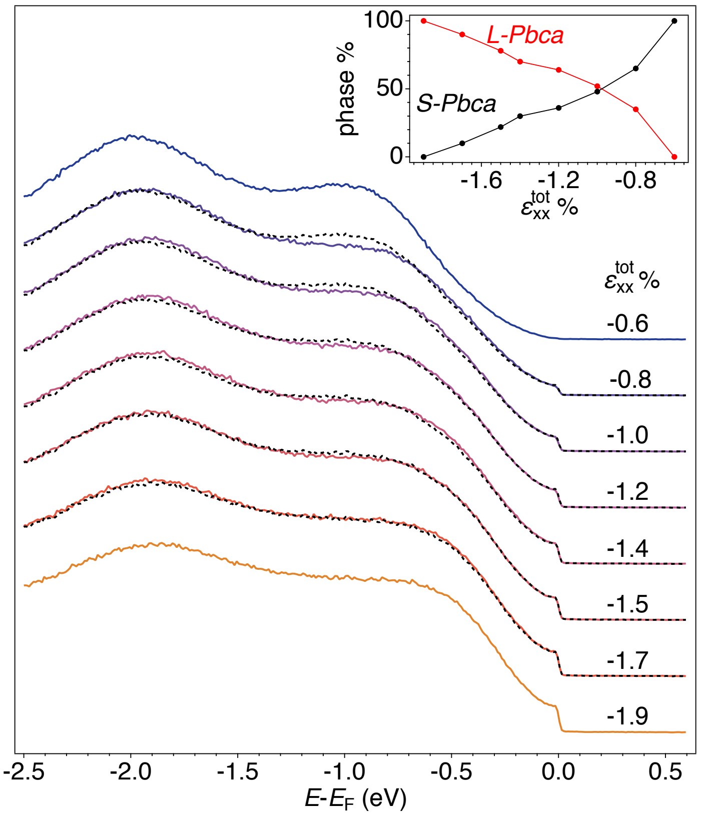

Supplementary Figure 4. Strain dependent angle-integrated EDCs. Colored lines are raw data reproduced from Fig. 2 of the main text. Dashed black lines are linear combinations of the spectra at $\epsilon_{xx} = -0.6\%$ and -1.9%, which are representative of the *L-Pbca* and *S-Pbca* phases, respectively. The coefficients of the linear combinations are given in the inset.

Supplementary References

* New permanent address: Deutsches Elektronen-Synchrotron DESY, Photon Science, Hamburg, 22607, Germany

† Correspondence and requests for materials should be addressed to F.B. (Felix.Baumberger@unige.ch)

[1] Friedt, O. *et al.* Structural and magnetic aspects of the metal-insulator transition in $Ca_{2-x}Sr_xRuO_4$. *Phys. Rev. B* **63**, 174432 (2001). URL `https://link.aps.org/doi/10.1103/PhysRevB.63.174432`.

[2] Takagi, H. *et al.* Systematic evolution of temperature-dependent resistivity in $La_{2-x}Sr_xCuO_4$. *Phys. Rev. Lett.* **69**, 2975–2978 (1992). URL `https://link.aps.org/doi/10.1103/PhysRevLett.69.2975`.

[3] De La Torre, A. *et al.* Collapse of the Mott Gap and Emergence of a Nodal Liquid in Lightly Doped Sr_2IrO_4. *Phys. Rev. Lett.* **115**, 176402 (2015). URL `https://link.aps.org/doi/10.1103/PhysRevLett.115.176402`.

[4] Cheggour, N. & Hampshire, D. P. A probe for investigating the effects of temperature, strain, and magnetic field on transport critical currents in superconducting wires and tapes. *Review of Scientific Instruments* **71**, 4521–4530 (2000). URL `http://aip.scitation.org/doi/abs/10.1063/1.1324734`.
