# Supplementary material for: In situ strain tuning of the metal-insulator-transition of Ca2RuO4 in angle-resolved photoemission experiments
Source: Nat Commun. 2018 Oct 31;9:4535. doi: 10.1038/s41467-018-06945-0 (PMC6208396; doi:10.1038/s41467-018-06945-0)
Supplement: Supplementary file 3 — Description of Additional Supplementary Files [file 41467_2018_6945_MOESM3_ESM.pdf]

Description of Additional Supplementary Files

File Name: Supplementary Data 1

Description: Supplementary Data file 1 contains the CIF file reporting the crystal structure determined by single crystal neutron diffraction of $Ca_{2-x}La_xRuO_4$ with x = 0.11 measured at 4 K.

File Name: Supplementary Data 2

Description: Supplementary Data file 2 contains the CIF file reporting the crystal structure determined by single crystal neutron diffraction of $Ca_{2-x}La_xRuO_4$ with x = 0.07 measured at 10 K.

File Name: Supplementary Data 3

Description: Supplementary Data file 3 contains the CIF file reporting the crystal structure determined by single crystal neutron diffraction of $Ca_{2-x}La_xRuO_4$ with x = 0.07 measured at 300 K.

File Name: Supplementary Data 4

Description: Supplementary Data file 4 contains the CIF file reporting the crystal structure determined by single crystal neutron diffraction of $Ca_{2-x}Pr_xRuO_4$ with x = 0.07 measured at 10 K.

File Name: Supplementary Data 5

Description: Supplementary Data file 5 contains the CIF file reporting the crystal structure determined by single crystal neutron diffraction of $Ca_{2-x}Pr_xRuO_4$ with x = 0.07 measured at 300 K.

File Name: Supplementary Data 6

Description: Supplementary Data file 6 contains the CIF file reporting the crystal structure determined by single crystal neutron diffraction of $Ca_{2-x}La_xRuO_4$ with x = 0.04 measured at 10 K.

File Name: Supplementary Data 7

Description: Supplementary Data file 7 contains the CIF file reporting the crystal structure determined by single crystal neutron diffraction of $Ca_{2-x}La_xRuO_4$ with x = 0.04 measured at 300 K.

File Name: Supplementary Data 8

Description: Supplementary Data file 8 contains the CIF file reporting the crystal structure determined by single crystal neutron diffraction of $Ca_{2-x}Nd_xRuO_4$ with x = 0.04 measured at 300 K.

File Name: Supplementary Data 9

Description: Supplementary Data file 9 contains the CIF file reporting the crystal structure determined by single crystal neutron diffraction of $Ca_{2-x}Nd_xRuO_4$ with x = 0.04 measured at 10 K.

File Name: Supplementary Data 10
Description: Supplementary Data file 10 contains the CIF file reporting the crystal structure determined by single crystal neutron diffraction of $Ca_{2-x}Pr_xRuO_4$ with x = 0.04 measured at 10 K.

File Name: Supplementary Data 11
Description: Supplementary Data file 11 contains the CIF file reporting the crystal structure determined by single crystal neutron diffraction of $Ca_{2-x}Pr_xRuO_4$ with x = 0.04 measured at 300 K.

File Name: Supplementary Data 12
Description: Supplementary Data file 12 contains the CIF file reporting the crystal structure determined by single crystal neutron diffraction of Ca_2RuO_4 measured at 10 K.

File Name: Supplementary Data 13
Description: Supplementary Data file 13 contains the CIF file reporting the crystal structure determined by single crystal neutron diffraction of Ca_2RuO_4 measured at 300 K.
